# Recovery of Biologically Treated Textile Wastewater by Ozonation and Subsequent Bipolar Membrane Electrodialysis Process

**DOI:** 10.3390/membranes11110900

**Published:** 2021-11-21

**Authors:** Burak Yuzer, Huseyin Selcuk

**Affiliations:** Department of Environmental Engineering, Faculty of Engineering, Istanbul University-Cerrahpasa, Avcilar, Istanbul 34320, Turkey; hselcuk@iuc.edu.tr

**Keywords:** bipolar membrane electrodialysis, ozonation, textile wastewater, desalination

## Abstract

The Bipolar Membrane Electrodialysis process (BPMED) can produce valuable chemicals such as acid (HCl, H_2_SO_4_, etc.) and base (NaOH) from saline and brackish waters under the influence of an electrical field. In this study, BPMED was used to recover wastewater and salt in biologically treated textile wastewater (BTTWW). BPMED process, with and without pre-treatment (softening and ozonation), was evaluated under different operational conditions. Water quality parameters (color, remaining total organic carbon, hardness, etc.) in the acid, base and filtrated effluents of the BPMED process were evaluated for acid, base, and wastewater reuse purposes. Ozone oxidation decreased 90% of color and 37% of chemical oxygen demand (COD) in BTTWW. As a result, dye fouling on the anion exchange membrane of the BPMED process was reduced. Subsequently, over 90% desalination efficiency was achieved in a shorter period. Generated acid, base, and effluent wastewater of the BPMED process were found to be reusable in wet textile processes. Results indicated that pre-ozonation and subsequent BPMED membrane systems might be a promising solution in converging to a zero discharge approach in the textile industry.

## 1. Introduction

Since the textile industry consumes large amounts of water and generates wastewater with high content of various organic, inorganic and toxic contaminants, it has been one of the most studied sectors [1,2,3]. Conventional treatment methods alone are not able to supply sufficient treatment to remove all pollutants from wastewater. Advanced treatment methods such as advanced oxidation processes (AOPs) and membrane filtration processes (MFPs) may follow conventional treatment methods to remove all organic and toxic pollutants [4,5,6]. However, the salt constituent of textile wastewater (60–100 g/L) remains a problem to deal with [7]. It is possible to remove salt content from wastewater via high pressure-driven membrane filtration processes such as nanofiltration (NF) [8] and reverse osmosis (RO) [9]. On the other hand, membrane fouling, chemical cleaning requirement, brine generation, operational and initial investment cost of these processes remain as a problem. The electrodialysis (ED) process has gained importance in recent years due to its high desalination capacity of waters with high salt content. ED is an electrochemical separation process of anions and cations with the use of semipermeable ion exchange membranes under applied electrical potential [10]. Ion exchange membranes are critical components in the ED process since anion exchange membranes have positively charged groups and cation exchange membranes have negatively charged groups that are fixed to the polymer matrix [11].

The ED process has many advantages when compared to high pressure-driven membrane filtration processes as it requires neither high pressure [12] nor chemicals for operation and membrane protection, which translates into lower operating and maintenance costs. However, as the aim is to produce a desalinated/treated stream (diluate), an ion-rich stream (concentrate) is also produced. The main problem for most membrane processes emerges as the management of concentrate stream and the ED is no exception. To circumvent the concentrate production issue, integration of bipolar membranes in electrodialysis membrane stacks comes as a solution. A bipolar membrane is composed of an anion exchange membrane and a cation exchange membrane that is put together. Once electrical potential is applied H^+^ and OH^-^ ions are produced as a result of water dissociation that occurs in a thin (4–5 nm) transition region between the cation- and anion-exchange layers of the bipolar membrane [11]. This unique property is used to produce acid and alkali streams when combined with the transferred ions from the desalinated stream. Therefore, bipolar membrane electrodialysis (BPMED) achieves not only desalination but also the production of acidic and alkaline streams that can be used in the industry.

Successful application of the BPMED process has been reported by Badruzzaman et al. [13]. In their study, the BPMED process has been integrated into the membrane system includes RO and electrochlorination processes to treat the concentrate stream of the RO process. In addition, Readi et al. [14] used bipolar membranes for internal pH control in the electrodialysis of amino acids. In another study, Petrov et al. [15] applied the BPMED process to neutralize industrial effluents before being discharged. Furthermore, the BPMED process was used for energy-efficient malic acid production by Luo et al. [16]. Recovery of the valuable chemicals from a waste solution is also possible by using the BPMED process. Gao et al. [17] have proposed the BPMED process to convert waste lithium bromide into lithium hydroxide while producing valuable hydrobromic acid as a by-product. For the same purpose, Kuldeep et al. [18] have recycled sulfate in the metallurgical industries through a feed and bleed BPMED process. 

In addition to the use of the BPMED process in the industries for various applications, desalination of the textile wastewater can be achieved with this process while producing acid and base from the salt content of the wastewater [19]. However, the textile wastewater has to be pretreated beforehand, to increase ion-exchange membranes’ service time and BPMED process efficiency. Otherwise, ion-exchange membranes may be clogged with organic matter, dye solution, and hardness causing divalent cations (Ca^2+^, Mg^2+^) [20,21]. Lin et al. [22] have explored the textile wastewater for resource recovery from a dye/NaCl stream by NF/BPMED process. The NF permeate had high salt content and no color observed in it, was treated by the BPMED to produce acid, base, and pure water without contamination of the AEM. Yao et al. [23] have proposed a treatment system including reverse osmosis (RO), electrochemical oxidation (EO), and BPMED processes to treat dyeing wastewater with a zero discharge aim. The wastewater was first treated with the RO process. Then, the concentrated stream produced in the RO system was pre-treated with the EO process and then desalinated by the BPMED process. They have achieved a 97% recovery ratio wastewater with the proposed system. In another study, Lafi et al. [24] have used the ultrafiltration (UF) process as a pretreatment method of the ED process and achieved 97% conductivity removal. 

Most studies generally have focused on high-pressure-driven membrane filtration processes as a pretreatment method for the BPMED process. However, the applicability and effect of other treatment methods on the BPMED process still are unknown. In the literature, there are many examples of textile wastewater treatment with the ozonation process to remove organic matter and dye content [25,26,27,28,29]. Malik et al. [26] have reviewed the literature and reported that color removal efficiencies between 90–100% ratios can be achieved for textile wastewater by using the ozonation process. Similarly, Bilinska and Gmurek have listed the COD removal efficiencies in the range of 80–100% for textile wastewater [28]. Moreover, the ion-exchange process is a proven method to remove hardness-causing ions from water and wastewater. Dahmani et al. [30] have achieved 99% and 68% removal efficiencies of Ca^2+^ and Mg^2+^ ions, respectively. Ilhan et al. [31] have treated landfill leachate via the BPMED process, using the cation-exchange process as a pretreatment method to remove Ca^2+^ and Mg^2+^ ions and have achieved more than 93.5% and 99% treatment efficiencies, respectively. 

In this scope, instead of using high-pressure-driven membrane filtration processes, the ozonation and ion-exchange resin processes might be the effective pretreatment methods to remove organic matter, color, and divalent cations from the wastewater. When used together, they not only have high ion, organics, and color removal efficiencies but also circumvent sludge production. For the first time in this study, the applicability and effect of the ozonation process as a pretreatment method for the BPMED process were investigated. Various process parameters of the BPMED system such as pH of the feed solution, energy consumption, current density, and conductivity removal efficiency were examined. 

## 2. Materials and Methods

**Figure 1:**
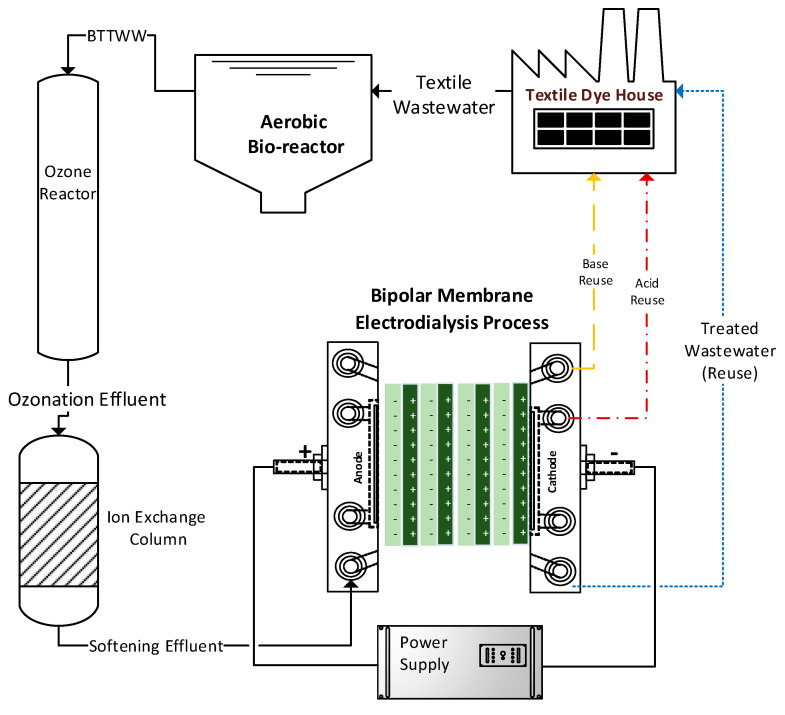
Schematic representation of the proposed treatment process for the textile wastewater.

Biologically treated textile wastewater (BTTWW) samples that were taken from the effluent of a full-scale biological treatment plant were filtered and then oxidized by ozone gas to remove color. Then, hardness-causing divalent cations (Ca^2+^ and Mg^2+^) were removed from wastewater via an ion-exchange column filled with Na-based ion exchange resin. Effluent from the ion-exchange column was fed to the BPMED reactor [32]. The main aim of the proposed treatment scheme was to achieve water recovery along with acid and alkali production (Figure 1). 

### 2.1. Textile Wastewater

Textile wastewater samples were taken from Gümüşsu Arıtma Inc. located in Denizli, Turkey. The facility has a full-scale biological treatment plant and textile wastewater samples were taken directly from the effluent stream. Wastewater quality parameters such as alkalinity, COD, hardness, and ozone concentration in wastewater were measured triplicate according to Standard Methods, APHA [33], and average values were recorded. Triplicate color measurements were carried out at 540 nm as Pt/Co along with UV-Vis scans in the wavelength range of 200–700 nm by using UV–Vis spectrophotometer (DR 5000, Hach, Düsseldorf, Germany). Total organic carbon (TOC) measurements were carried out by using the Shimadzu TOC-CPN TOC analyzer. This device takes triplicate measurements and gives the average value of the TOC concentration. A multi-parameter pH and conductivity control system (SC1000, Hach, Düsseldorf, Germany) was employed for continuous pH and conductivity measurements during the BPMED system operation. Produced acid and base molar concentrations were calculated from their pH values.

### 2.2. Ozonation Process

Sander Model 300.5 ozone generator was utilized to generate ozone gas from the pure oxygen gas with a supplied flow rate of 500 NL/h. The ozone generator worked at 250 mA electrical current and generated 4.5 g O_3_/h with a concentration of 14 g O_3_/m^3^ [34]. Ozone treatment was applied to BTTWW samples until the color of wastewater has been removed. Ozone trap using gas washing bottles with 20 g/L KI solution was set up at the end of the ozone contact reactor to analyze ozone production rate and reacted ozone concentration, as explained by Standard Methods 2350 D, APHA [33]. Initially, ozone gas was supplied directly to the wastewater to determine the time of the complete color removal which was achieved in 25 min. Then, ozone gas was given to the ozone trap filled with KI solution for different periods (5, 15, 25 min) to calculate the generated ozone gas amount. Finally, ozone analyses were performed according to Standard Methods 2350 D, APHA [33]. The same procedure was applied after the ozonation of BTTWW to analyze the ozone reaction rate inside the ozone contact reactor. 

### 2.3. Water Softening via Ion-Exchange Resins

Hardness-causing divalent ions (Ca^2+^ and Mg^2+^) may cause calcification on ion-exchange membrane surfaces in the BPMED process. Therefore, hardness should be removed to prevent membrane scaling. Ion exchange resin is an effective way to remove hardness since there is no sludge production in the process. As a result, BTTWW was passed through a laboratory-scale ion-exchange column filled with Na-based cationic ion exchange resins (Purolite^®^ C100). The typical characteristics of the ion-exchange resin are given in Table 1. 

### 2.4. Bipolar Membrane Electrodialysis Process

BPMED unit (ED 64-4 Cell, PCCell GmbH Company, Heusweiler, Germany) was operated in batch mode with a three-compartment configuration. Polypropylene containers were used to feed acid, base, wastewater, and electrolyte streams to the BPMED cell. Common properties of BPMED cell unit and ion exchange membranes are listed in Table 2. Anion exchange membrane (PC-SA) and standard cation exchange membrane (PC-SK) were utilized in all experiments. Spacers were installed between two membranes to facilitate liquid flow inside the BPMED cell. 

**Figure 2:**
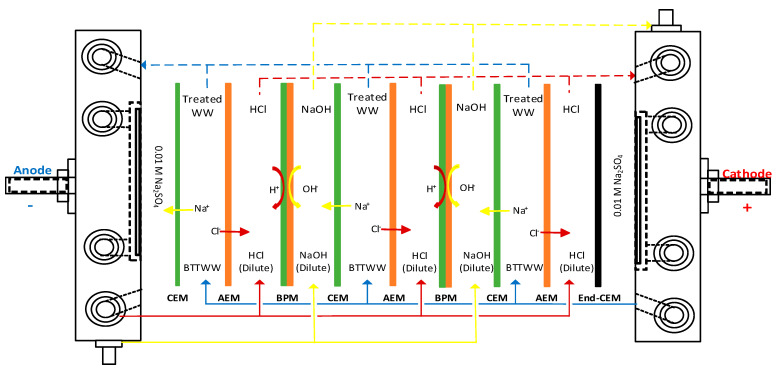
Schematic illustration of BPMED cell operation (CEM: cation exchange membrane, AEM: anion exchange membrane, BPM: bipolar membrane, WW: wastewater).

For every single experimental run was carried out in the batch mode, the applied potential was kept constant, and the current was changed concerning wastewater conductivity. Centrifugal pumps with flow rates in the range of 4–8 L/h were utilized to supply wastewater and electrolytes to the BPMED system. Hyder et al. [36] recommended keeping the transmembrane pressure between all compartments lower than 1.4 kPa to stabilize the electrodialysis system. Similarly, the manufacturer recommended the transmembrane pressure should be kept as zero during the BPMED process run to prevent water transfer through ion-exchange membranes. Thus, the flow rates of the solutions have been adjusted to maintain the same pressure in all compartments to keep transmembrane pressure as zero by using pressure gauges mounted to pipelines. Online pH and conductivity values of all compartments were saved to a computer continuously. Since transmembrane pressure should be zero between the anion and cation-exchange membranes to prevent water transfer between the compartments, the control of pressure was carried out via pressure gauges mounted on pipelines. In all experiments, 0.01 M Na_2_SO_4_ was used as the electrolyte solution for anode and cathode sides, and 0.01 M HCl and 0.01 M NaOH solution were prepared to supply initial electrical conductivity in acid and base compartments, respectively (Figure 2).

The removal efficiency of ions from BTTWW was calculated using the following expression:

(1) $$RE,\%=\left(\frac{C_{0}-C_{t}}{C_{0}}\right)\times 100\;$$

where; *C*_0_ is the initial conductivity of the BTTWW and *C_t_* is the conductivity value of BTTWW at time t.

The ion separation rate in an ideal BPMED process is proportional to the current density through the BPMED stack. Current density (*i*) of the BPMED process was calculated by using the following formula [36]:

(2) $$i=\frac{I}{A_{mem}}$$

where; *I* is the electrical current (mA), and *A_mem_* is the area of a single membrane (cm^2^).

## 3. Results and Discussion

Well-designed pretreatment methods are essential to prevent membrane fouling and extend membranes’ lifetime. Organic matter, dye solution, and hardness-causing cations are the main factors that clog membranes in the BPMED system [20,21]. While biological treatment removes organic matter, subsequent ozone oxidation enhances organic matter and dye removal from the wastewater (Table 3). 

**Figure 3:**
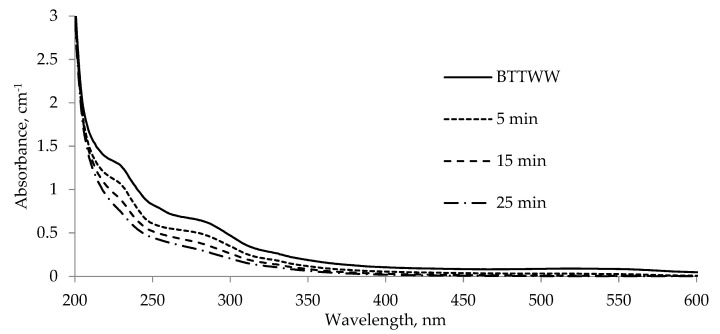
BTTWW color and absorbance values change with respect to ozonation time.

Conventional inorganic coagulants such as alum and Fe^2+^, are not sufficient for the removal of soluble anionic dyes and high volume sludge production is the main disadvantage of inorganic coagulation [2]. Organic polymers have therefore been developed for color removal and, in general, they offer significantly improved performance with low investment cost [1]. However, they have the same disadvantages as their inorganic counterparts: the production of chemical sludge. Ozonation, being an advanced oxidation technique, can remove color without producing sludge. Still, its investment and energy costs are high. So, generally traditional methods for dealing with textile wastewater include various combinations of biological, physical, and chemical treatment methods [4]. In terms of desalination and reuse of textile wastewaters, anionic or cationic residuals of organic or inorganic coagulants may cause clogging and fouling problems on the cationic/anionic membrane surface, and also the salinity of wastewater may increase depending on the coagulant dose. Ozone doesn’t increase wastewater salinity and leave residuals, causing membrane fouling problems. In this study, ozone gas was supplied to BTTWW until complete color removal was achieved. UV-vis scans for pre- and post-ozonation BTTWW samples were given in Figure 3 (black curves). To evaluate ozonation efficiency, samples at 5 min, 15 min, and 25 min were taken from the ozone reactor and analyzed for color. At the end of 25 min, almost 94% of color was removed (Figure 3). 

**Figure 4:**
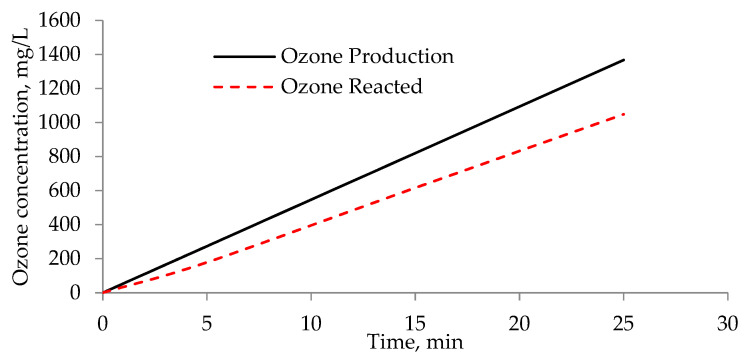
Ozone production and consumption rate in the ozone reactor.

It was also observed that percentage of unreacted ozone leaving the reactor decreased with prolonged reaction time. While 23.3% of the ozone gas left the reactor unreacted at 25 min, it was 34.6% at 5 min (Figure 4). At 25 min reaction time, approximately 1000 mg/L ozone reacted with BTTWW, achieving 40% COD and 15% TOC removal rates. Although the highest removal rates were achieved at 25 min, 15-min ozonation achieved sufficient color removal such that its effluent is safe to feed to the BPMED process.

During the BPMED process, Ca^2+^ and Mg^2+^ cause calcification on the ion-exchange membrane surface and reduce the desalination efficiency of the BPMED process [31]. Removal of divalent cations is also essential while producing acid and base from wastewater due to reuse requirements in wet textile processes. Using ion-exchange resins for hardness removal from process water is a common practice in the textile sector. Regeneration wastewater of the softening process is the only hardness-causing source in textile wastewater as inorganic divalent ions are not used in wet textile processes. Once regeneration wastewater from softening process is mixed with textile wastewater, textile wastewater entering the biological treatment plant ends up containing hardness. Separation of softening regeneration wastewater is merely an infrastructure issue and once it is carried out, hardness will cease to be an issue for any membrane treatment application, including ED. The ozonated textile wastewater was passed from a Na-based ion-exchange column to remove hardness, as a result, the hardness of the wastewater was decreased from 545 to <5 mg CaCO_3_/L. 

### 3.1. Bipolar Membrane Electrodialysis Process

**Figure 5:**
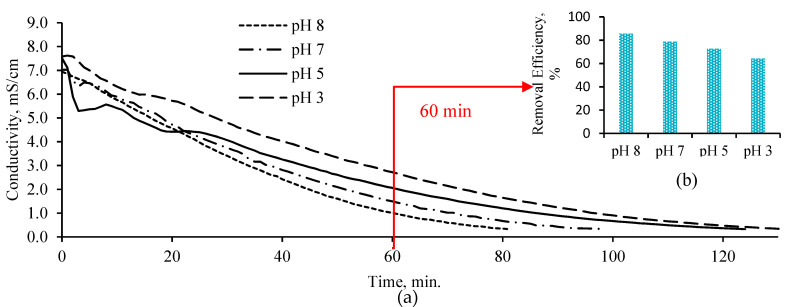
Conductivity values for BPMED effluent at various initial pH values (**a**) and conductivity removal efficiency at 60 min working period (**b**).

BPMED process was operated in batch mode and constant potential difference (15 V) was applied between anode and cathode for the whole process time. As desalination of BTTWW proceeded, the conductivity of BTTWW decreased, which in turn caused an increase in resistivity and decrease in current. The initial pH of BTTWW was adjusted to 3, 5, 7, and 8 to evaluate the pH effect. Petrov et al. [15] have investigated the effect of the feed solution pH value of the industrial wastewater on the BPMED process. Their results have shown that above pH 11.0 and below 3.0, even at high current densities (*i* > 20 A/m^2^), the BPMED process did not provide efficient treatment. A similar result has been seen in this study, while low pH leads to a lower ion transfer rate (Figure 5a). Required desalination time for BTTWW increased with the decrease in initial pH. Comparison of conductivity removal efficiencies for pH values at 60 min showed that the highest removal efficiency (85%) was achieved for initial pH 8 (Figure 5b). These findings contrast previous results reported in the literature [21]. Berkessa et al. [21] have revealed that the BPMED process achieved lower stack voltage and stable operation at pH 3 compared to pH 7 and 11. This situation was explained by the fouling effect of the dye inside the feed solution on the ion-exchange membranes. However, in this study, the dye solution in the textile wastewater was removed by using ozonation and did not affect the BPMED process efficiency. Strathmann [12] has indicated that increasing acid and base concentration cause a decrease in the permselectivity of the ion-exchange layers of the bipolar membrane due to the Donnan exclusion effect which results in salt leakage. In addition, current utilization is affected by salt leakage through the bipolar membrane. Similarly, at higher acidic and alkaline conditions H^+^ ions can permeate the anion-exchange membrane while OH^−^ ion can permeate the cation-exchange membrane. The net result becomes neutralization of H^+^ and OH^−^ -ions generated in the bipolar membrane and thus reduction of current utilization. The decrease in removal efficiencies of the BPMED process with decreasing the initial pH value of the feed solution can be attributed to the reduction of current utilization.

Since the highest conductivity removal efficiency was achieved when the initial pH of the wastewater was 8, this pH value is also effective for ozonation treatment. Malik et al. [26] have reported that the efficiency of the ozonation process depends on the pH of the solution because a high amount of hydroxyl radical which is a strong oxidant occurs at alkaline conditions. In addition, Morali et al. [29] have investigated the effect of pH on the ozonation kinetics. It was revealed that 94.7% color removal efficiency has been achieved at pH 9.3. These results show that ozonation and bipolar membrane electrodialysis processes provide more effective treatment at high pH values. It is seen that textile wastewater can be treated directly by ozonation and BPMED processes without pH adjustment. In this case, the operating cost of the system may decrease as no chemicals will be used for pH adjustment.

The overall removal efficiencies for COD, color, and conductivity parameters of ozonation and BPMED system were calculated as 62%, 97%, and 96%, respectively. Keskin et al. [37] have compared the removal efficiencies of membrane processes for textile wastewater treatment. The ultrafiltration (UF) process has provided low conductivity removal however, COD and color removal efficiencies were given as 64–76%, and >75%, respectively. Keskin et al. [37] have also reported that when UF and RO processes were applied to textile wastewater after biological treatment in another study, COD, color, and conductivity removal efficiencies were obtained as 76%, 95%, and 99%, respectively. It can be concluded that similar treatment efficiencies with high-pressure-driven membrane processes, can be obtained by using the ozonation and subsequent BPMED processes.

**Figure 6:**
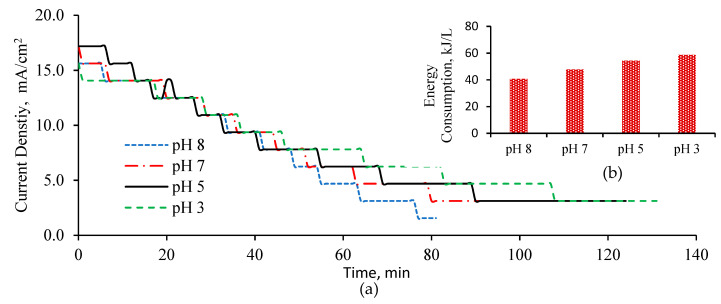
Current density values of BPMED system operated with different initial pH values (**a**), energy consumption for desalination of 1 L BTTWW (**b**).

Operating current densities for different initial pH values of BTTWW samples started from 15 mA/cm^2^ for the pH values of 8 and 3 and 17 mA/cm^2^ for the pH values of 7 and 5, respectively, and dropped to 3.12 mA/cm^2^ at the end of the process (Figure 6a). The exhaustion of the ions in the BTTWW led to an increase in the resistance of the feed compartment thus, this caused decrease in the current densities during the BPMED process. As a result, more energy was consumed to transfer residual ions in the feed wastewater. The energy consumption of the BPMED process to desalinate one-liter BTTWW under different initial pH values of the solution is given in Figure 6b. 

When the initial pH value of BTTWW was adjusted to 8, energy consumption was calculated as the lowest compared to other pH values, since desalination took place in a shorter time at pH 8. The current density of the BPMED process affects the energy costs and required membrane area. The energy costs of the BPMED process increase linearly while the required membrane area decreases in a hyperbolic function with increasing current density [38]. Therefore, to achieve minimum desalination cost the current density of the BPMED process should be well chosen. Chandramowleeswaran et al. [39] reported that the recommended current density was 3.6–4.8 mA/cm^2^ for the treatment of textile wastewater with 7000 mg/L total dissolved solid concentration. Conversely, Schoeman [40] has studied electrodialysis with high current densities between 20–120 mA/cm^2^ and reported that membrane fouling was experienced at higher current densities. In addition, optimized current density for the bipolar membrane electrodialysis process that was treating sodium acetate waste residue was reported from Xue et al. [41] as 50 mA/cm^2^. 

### 3.2. Fate and Transport of Organic Matter

**Figure 7:**
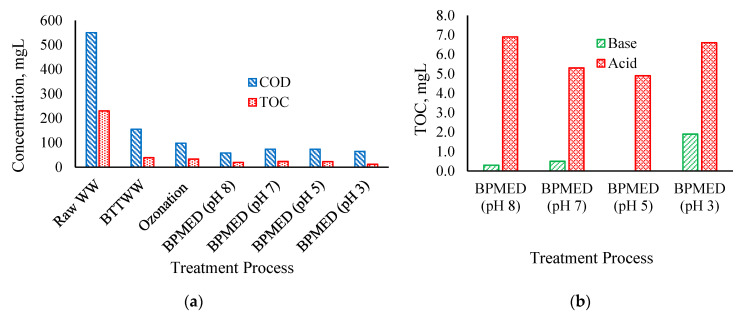
COD and TOC concentrations after each treatment process (**a**), TOC concentrations of acid and base compartments (**b**).

Even though the BPMED system was designed for ion removal from wastewater, COD and TOC removals were also observed. Maximum 40% COD removal and 50% TOC removal were observed at pH 8 (Figure 7a). Accumulation of organic matter on the membrane surface may be an explanation for this phenomenon at the higher initial pH of the feed solution. Since the high pH value of the solution facilitates gel formation and/or precipitation on the membrane surface, the solution pH plays a significant role in membrane fouling [20,21]. Although the removal of organic matter may appear to be adding to the merit of the system, it will cause membrane fouling and shorten the lifetime of membranes in the long run [19]. Hansima et al. [20] have reviewed the fouling of ion-exchange membranes and reported the factors affecting IEM fouling by organic matters as molecular weight fractions, feed solution pH, organic matter solubility, molecular charge, macromolecular structure, surface roughness, and pore size of nanochannels. The fouling of the IEMs adversely affects the desalination efficiency of the BPMED process [42]. Efficient removal of the organic matter from the feed solution is required to prevent organic fouling of the IEMs. In addition, some small portions of an organic matter passed through the ion-exchange membranes, to acid and base compartments. The concentration of organic matter in the acid compartment was higher than the base compartment, for which can be concluded that organic matter was negatively charged (Figure 7b).

**Figure 8:**
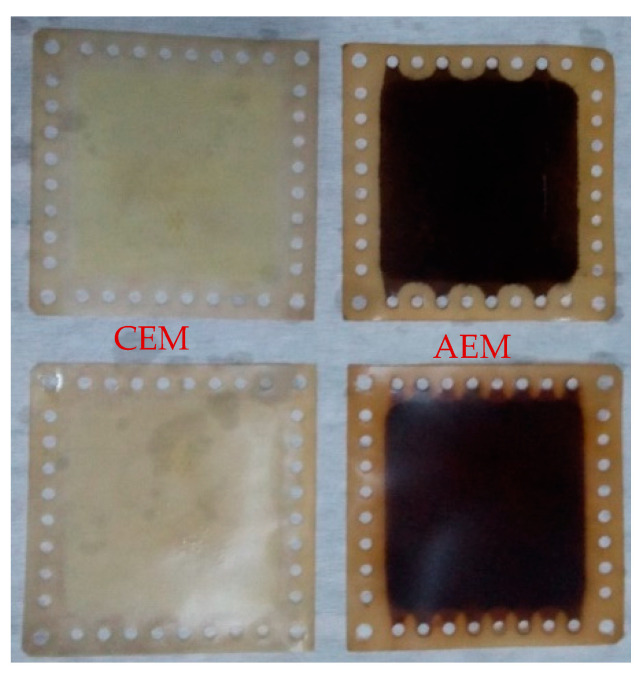
Ion exchange membranes after the BPMED process.

In addition, the BPMED reactor has been tested without applying color removal from the wastewater and it was seen that only AEMs were stained with dye solution remained in the wastewater. The images of the used ion-exchange membranes given in Figure 8 revealed that the dye solution is negatively charged and accumulated on the AEM. This result was consistent with the data given in Figure 7b and showed that there would be no fouling due to the organic content of BTTWW on CEM. Ma et al. [43] have modified cation-exchange membranes by using polyethyleneimine, titanium dioxide nanoparticles, and graphene oxide nanosheets to solve the organic fouling problem of the membranes. It was reported that the superior antifouling capacity of the nanocomposite membranes was achieved over a 100-h operation. Apart from this Berkessa et al. [21] have claimed that maintaining the zeta potential of Remazol Brilliant Blue R in textile wastewater above −25 mV may mitigate fouling of AEM during the BMED process. In addition, it could be stated that when the initial pH was adjusted to 3, the charged organic matters passed through the bipolar membrane to the base solution due to the above-mentioned Donnan exclusion effect similar to salt leakage [12]. In this system, the organic mass balance could not be written since some portion of the organic content was oxidized, and some portion was accumulated on ion-exchange membranes.

### 3.3. Acid and Base Production via Bipolar Membrane Electrodialysis Process

**Figure 9:**
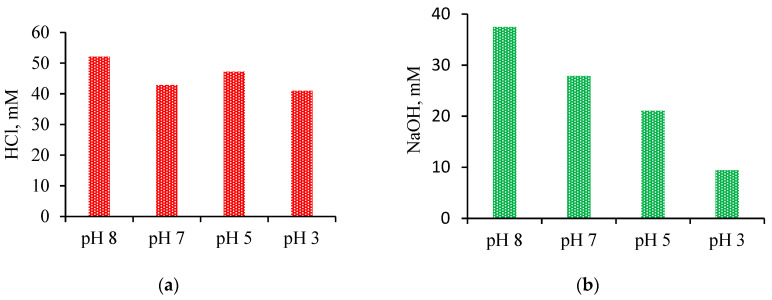
Acid (**a**) and base production (**b**) with different initial pH values at 60 min.

The highest acid production was established at pH 8 with 52 mM HCl production at 8 mS/cm conductivity value by using the BPMED process (Figure 9a). On the other hand, when base production rates were compared, it was evident that base production was adversely affected by pH changes. At lower initial pH values, NaOH production almost stopped (Figure 9b); however, it increased when the initial pH value was adjusted to 8. The maximum amount of NaOH production was 37 mM at pH 8.

## 4. Conclusions

The BPMED process is a promising technology for the treatment of textile wastewater as it produces acid and base from the salt content of the wastewater and it does not produce any waste stream that needs further treatment. However, fouling of the ion-exchange membranes due to dye solution, organic matter, and hardness-causing ions is the main drawback of the process. The ozonation process has been successfully applied and provided 90% color removal efficiency from the BTTWW. The ion-exchange resin process was a vital step that prevented scaling caused by Ca^2+^ and Mg^2+^ on membranes by removing them from wastewater with a 99% efficiency. As a result, it can be concluded that the applied ozonation and ion-exchange resin processes have prevented the fouling of the ion-exchange membranes. In addition, the effect of the initial pH value of the feed wastewater on the BPMED process was investigated and the results have reflected that the best performance of the BPMED process was achieved at pH 8. Considering that high amounts of hydroxyl radicals are produced in the ozonation process at high pH values, the BTTWW can be treated without the requirement of pH adjustment by using the BPMED process after ozonation. The treatment system composed of ozonation, ion-exchange resin, and BPMED processes has the removal efficiencies for COD, color, and conductivity parameters were calculated as 62%, 97%, and 96%, respectively. In addition, the BPMED process has produced 52 mM HCl and 37 mM NaOH. Therefore, the BPMED process offered an efficient system for the desalination of textile wastewaters.

## Figures and Tables

**Table 1:** The typical characteristics of the ion-exchange resin [35].

Polymer Structure	Gel Polystyrene Crosslinked with Divinylbenzene
Appearance	Spherical Beads
Functional Group	Sulfonic Acid
Ionic Form	Na^+^ form
Total Capacity (min.)	2.0 eq/L (Na^+^ form)
Reversible Swelling, Na^+^ → H^+^ (max.)	9%
Specific Gravity	1.29
Temperature Limit	120 °C

**Table 2:** Common properties of BPMED cell unit and ion exchange membranes.

Anode	Pt/Ir–Coated Titanium	
Cathode	V4A Steel	
Electrode housing material	Polypropylene	
Maximum current	5A	
Maximum voltage	30 V/cell	
Nominal flow rate	4–8 L/h	
Membrane Type	Anion exchange	Cation exchange
Functional group	Strong basic	Strong acidic
	Ammonium	Sulfonic acid
Permselectivity KCl (0.1/0.5 N) Acid (0.7/3 N)	>0.95	>0.95
Resistivity, W.cm^2^	≈1.8	≈2.5
Active membrane area, cm^2^	64	64
Water content, (wt. %)	≈14	≈9
Max operational temperature, °C	60	50
Thickness, µm	180–220	160–200
Membrane size, mm	110 × 110	110 × 110
Ionic form	Cl^−^	Na^+^

**Table 3:** Characterization of textile wastewater influent, effluent and post-ozonation streams.

Parameter	Influent	BTTWW	Ozonation	BPMED
pH	8.7	8.1	8.0	2.9–5.5
Conductivity, mS/cm	8.5	7.7	7.6	0.3–0.7
Color, PtCo	330	210	20	5–10
COD, mg/L	550	155	98	58–73
TOC, mg/L	230	38.5	32.8	20
Alkalinity, mg/L	1220	1420	1390	-
Total Hardness, mg CaCO_3_/L	570	550	545	-

## Data Availability

Not applicable.
